# Dyslipidemia in Acute Non-cardioembolic Ischemic Stroke Patients at a Tertiary Care Centre: A Descriptive Cross-sectional Study

**DOI:** 10.31729/jnma.7321

**Published:** 2022-03-31

**Authors:** Surendra Prasad Shah, Aashish Shrestha, Sirish Raj Pandey, Kaushal Sigdel, Namrata Sah, Sagar Panthi, Lila Bahadur Basnet

**Affiliations:** 1 Nobel Medical College Teaching Hospital, Biratnagar, Morang, Nepal; 2 Institute of Medicine, Tribhuvan University Teaching Hospital, Maharajgunj, Kathmandu, Nepal; 3 Nepal Cancer Hospital and Research Center, Lalitpur, Nepal; 4 Koshi Hospital, Biratnagar, Morang, Nepal; 5 Laxmipur Patari Healthpost, Siraha, Nepal; 6 B. P. Koirala Institute of Health Sciences, Dharan, Nepal; 7 Curative Service Division, Department of Health Services, Teku, Kathmandu, Nepal

**Keywords:** *cardioembolic stroke*, *dyslipidemia*, *ischemic stroke*, *lipid*, *lipoprotein*

## Abstract

**Introduction:**

Acute ischemic stroke is the second most common cause of death after ischemic heart disease worldwide and Nepal's top five diseases based on Disability-Adjusted Life Years. Dyslipidemia is a major risk factor for coronary heart disease but has an unclear role in the pathogenesis of ischemic stroke. The objective of this study was to find the prevalence of dyslipidemia in acute non-cardioembolic ischemic stroke patients at a tertiary care centre.

**Methods:**

A descriptive cross-sectional study was conducted among 76 patients with acute non-cardioembolic ischemic stroke admitted in the Neuromedicine unit of a tertiary care centre from August 2017 to July 2018. Ethical approval was obtained from the Institutional Review Committee of the same institute (Reference number: 478/2020). Patients underwent baseline investigations, including fasting lipid profile and Computed Tomography Scan/Magnetic Resonance Imaging head. Data were analysed using Statistical Package for the Social Sciences version 21.0. Point estimate at 95% Confidence Interval was calculated along with frequency and proportion for binary data.

**Results:**

The prevalence of dyslipidemia among the acute non-cardioembolic ischemic stroke patients was 35 (46.05%) (35.05-57.05 at 95% Confidence Interval) where high total cholesterol was diagnosed in 11 (31.43%), high triglycerides in 25 (71.43%), high low-density-lipoprotein in 10 (28.57%), and low high-density-lipoprotein in 11 (31.43%) patients.

**Conclusions:**

The prevalence of dyslipidemia among acute non-cardioembolic ischemic stroke patients at our tertiary care centre is higher than the similar studies done in similar settings.

## INTRODUCTION

Stroke is defined as an abrupt onset of neurologic deficit due to a focal vascular cause, ischemic or hemorrhagic.^1^ Stroke is the second most common cause of death behind cardiac ischemia globally according to World Health Organization (WHO) and is listed among the top five diseases based on disability-adjusted-life-years (DALYs) in Nepal.^2^

About 12.80% develop a second ischemic event within a year with an eight per cent annual risk for recurrence over three years.^3^ Studies have shown serum-lipids as a potential therapeutic target for risk reduction.^4-7^ The use of statins in patients with recent ischemic stroke has been found to reduce the incidence of its recurrence.^8^ A study in Kathmandu showed a three-fold rise in the incidence of dyslipidemia in stroke patients.^9^

The objective of this study was to find the prevalence of dyslipidemia among acute non-cardioembolic ischemic stroke patients at a tertiary care centre in Eastern Nepal.

## METHODS

A descriptive cross-sectional study was conducted among patients diagnosed with acute non-cardioembolic ischemic stroke visiting the Neuromedicine unit of the Nobel Medical College Teaching Hospital, Biratnagar, Nepal from 2017 August to 2018 July. Ethical approval was obtained from the Institutional Review Committee of the same institute (Reference number: 478/2020). Seventy-six patients aged 18 years and above, irrespective of gender, diagnosed clinically and/ or radiologically with acute non-cardioembolic ischemic stroke who consented to participate were included in the study. Patients with the previous history or recent electrocardiographic evidence of atrial fibrillation, echocardiography findings of a valvular, septal, or vegetative lesion, or any recent history of myocardial infarction, those presenting with a subacute or a past history of ischemic stroke, i.e., more than a week, those with hypoglycemia, hyponatremia, hemiplegic migraine, and seizure episode and those on lipid-lowering drugs were excluded from the study. A convenience sampling technique was used.

The sample size was calculated using the formula:

n = (Z^2^ × p × q) / e^2^

  = (1.96^2^ × 0.50 × 0.50) / 0.05^2^

  = 385

Where,

n = required sample size
Z = 1.96 at 95% Confidence Interval (CI)
p = prevalence taken as 50% for maximum sample size
q = 1-p
e = margin of error, 5%

But, the total number of acute ischemic stroke patients admitted in our hospital according to the last year data of 2016 was only 91.

Here n is Cochran's sample size recommendation, N is the finite population, and n' is the new adjusted sample size.

Adjusted Sample Size (n') = n / [1+ {(n-1) / N}]

    = 385 / [1 + (385 - 1) / 91)]

    = 74

However, 76 patients diagnosed with acute non-cardioembolic ischemic stroke were taken as sample size. The clinical definition of stroke has been consistent with the WHO definition, as rapidly developed clinical signs of focal or global disturbance of cerebral function, lasting for more than 24 hours with no apparent cause other than a vascular origin. Non-contrast computed tomography (NCCT) has been a widely used imaging technique to evaluate acute ischemic stroke. However, the task of identifying the early signs of acute ischemia and quantifying areas of brain involvement on NCCT scan may be missed due to the subtle or absent findings in earlier phases. So, the reliability of early ischemic stroke detection depends on clinical history, stroke window period, and the level of viewing the images.^10^ Meanwhile, confirmation through repeat CT-scan or MRI head later could not be done in all cases due to the socio economic condition of some patients who could not afford further expenses. Therefore, the diagnosis of ischemic stroke was made both clinically and/or radiologically wherever applicable.

Serum total cholesterol (TC), low-density lipoprotein (LDL) cholesterol, high-density lipoprotein (HDL) cholesterol, very-low-density lipoprotein (VLDL) cholesterol, and triglycerides (TG) were estimated in all cases. Blood samples were collected from patients after overnight fasting of 12 hours within 24 hours of admission.^11^ About 5-6 ml of blood was collected from each patient, and after retraction of a clot in about 4560 minutes, serum was separated, centrifuged to free from cells, and the clear serum was used for estimation of total cholesterol, triglycerides, HDL cholesterol, LDL cholesterol and VLDL cholesterol.

According to the Third Report of the National Cholesterol Education Program (NCEP) Adult Treatment Panel III,^12^ Dyslipidemia is characterised as fasting lipid profile values of

- LDL Cholesterol ≥130 mg/dl or
- Total Cholesterol ≥200 mg/dl or
- Triglycerides ≥150 mg/dl or
- HDL Cholesterol ≤40 mg/dl

Data were entered in Microsoft Excel 2016 and analysed using Statistical Package for the Social Sciences (SPSS) Version 21.0. Point estimate at 95% Confidence Interval and descriptive statistics were interpreted as frequency, percentage, or as mean and standard deviations.

## RESULTS

The prevalence of dyslipidemia among the acute non-cardioembolic ischemic stroke patients in our hospital was 35 (46.05%) (35.05-57.05% at 95% Confidence Interval). A high TC was found in 11 (31.43%), high TG in 25 (71.43%), high LDL cholesterol in 10 (28.57%), and low HDL cholesterol was found in 11 (31.43%) patients.

The mean age of the patients with acute non-cardioembolic ischemic stroke presenting with dyslipidemia was 62.83±11.13 years. Age-wise distribution revealed that 8 (22.86%) patients with dyslipidemia belonged to the 18-54 years age group and 27 (77.14%) to ≥55 years age group. Similarly, 2 (25.00%) from 18-54 years age group and 9 (33.33%) from ≥55 years age group had high TC while seven (87.50%) from 18-54 years age group and 18 (66.67%) from ≥55 years age group had high TG. Likewise, one (12.50%) from 18-54 years age group and 10 (37.04%) from ≥55 years age group had low HDL cholesterol while three (37.50%) from 18-54 years age group and seven (25.93%) from ≥55 years age group had high LDL cholesterol (Table 1).

**Table 1:** Lipid profile with different age groups.

Lipid profile	Age group	
	18-54 years (n= 8) n (%)	> 55 years (n= 27) n (%)
High TC	2 (25.00%)	9 (33.33%)
High TG	7 (87.50%)	18 (66.67%)
Low HDL	1 (12.50%)	10 (37.04%)
High LDL	3 (37.50%)	7 (25.93%)

**Figure 1:**
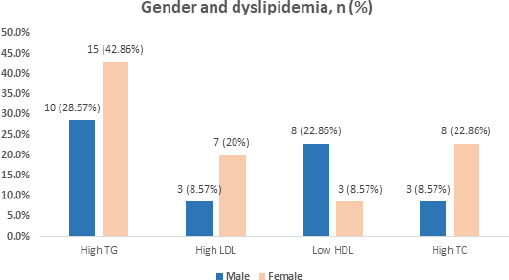
Dyslipidaemia in male and female patients (n= 35).

Gender-wise distribution revealed that the stroke patients with dyslipidemia included 20 (57.14%) females and 15 (42.86%) males. Similarly, 7 (22.86%) females and 3 (8.57%) males had high TC while 15 (42.86%) females and 10 (28.57%) males had high TG. Likewise, 7 (20.00%) females and 3 (8.57%) males had high LDL while 3 (8.57%) females and 8 (22.86%) males had low HDL (Figure 1).

## DISCUSSION

The prevalence of dyslipidemia among the acute non-cardioembolic stroke patients in our hospital was 46.05% with hypertriglyceridemia being the most associated culprit responsible for it. This high prevalence of dyslipidemia could be due to the sedentary lifestyle of people and the environment changes and is comparable to a study done by Thapa A, et al. among Nepalese patients with ischemic stroke during 2012-2015 where the prevalence of dyslipidemia was 34.10%.^9^ The same study by Thapa A, et al. also found a three-fold rise in the incidence of dyslipidemia in stroke patients but could not establish a positive correlation between dyslipidemia and ischemic stroke.^9^ Likewise, a study done by Olamoyegun MA, et al. in Nigeria showed an incidence of dyslipidemia (92.30%) in ischemic stroke patients, which is significantly higher as compared to our study which may be due to the fact that we included only non-cardioembolic ischemic stroke patients in our study.^13^

A study done in the United States in the mid-1990s among 27,937 healthy women ≥45 years of age revealed a significant association of all lipid levels, TC, LDL cholesterol, TC/HDL ratio, and non-HDL cholesterol with the increased risk of ischemic stroke.^6^ A randomised controlled trial carried out among 3680 ischemic stroke patients in Scotland, Canada, and the United States depicted elevated baselines TG/ HDL, and TC/HDL ratios as significant predictors for the future vascular risk following an ischemic stroke, while only the elevated TG/HDL ratio being related to the risk of recurrent stroke.^4^ Comparable to these findings, hypertriglyceridemia (71.43%) is among the greatest contributors to dyslipidemia in the patients in our study. Relatable findings with a large proportion of hypertriglyceridemia among the stroke patients were also reported in the independent studies by Maskey A, et al. in Nepal (23%) and Onwuegbuzie GA, et al. in Nigeria (43%).^14,15^ But, the study by Maskey A, et al. also concluded not to consider hypertriglyceridemia as a risk factor for ischemic stroke and recommended further studies to link this relation.^14^ However, hypertriglyceridemia being an important component of metabolic syndrome, the most important risk factor for prothrombotic state and atherosclerosis, both being strongly correlated to the increased risk of ischemic stroke.^16^ Thus, favouring the notion, lowering triglyceride could positively impact ischemic stroke risk reduction, supporting the initiation of statin treatment soon after a stroke or Transient Ischemic Attack and changing dietary habits for ischemic stroke prevention.

The findings from the Honolulu Heart Program study during 1965-68 among Japanese men (aged: 4568 years) revealed a significant three-fold higher risk of developing thromboembolic ischemic stroke with increasing low HDL compared to those with adequately high HDL levels (>60 mg/dl).^17^ This notion was supported by a case-control study done in Manhattan during 1993-97 where increased HDL levels were associated with reduced risk of ischemic stroke among the elderly which is well understood by the basic understanding of the physiology of HDL promoting extrahepatic cholesterol transport to the liver, thus lowering the serum cholesterol and preventing the ischemic stroke.^18^ But, low HDL pattern was less common among the stroke patients in our study (31.43%) as compared to other lipid profile findings, in contrary to the findings from Honolulu and Manhattan studies, which may be due to the variation in the sample size compared to these large studies and inclusion of only the non-cardioembolic ischemic stroke patients in our study. However, the analysis of 25 cohort studies from 196694 by Woodward M, et al. did not find any significant association of either HDL cholesterol or TC/HDL cholesterol ratio with ischemic stroke, contradicting the findings from other studies.^6,17-19^

Similarly, the Hisayama study by Imamura M, et al. among 2351 Japanese inhabitants aged ≥40 years of age showed the increased incidences of atherothrombotic and lacunar infarctions significantly with the increasing LDL cholesterol level but no such associations were observed for cardioembolic infarction, thus depicting a positive association of increased LDL with small artery stroke as high LDL is usually implicated in atherosclerosis.^20^ Contrary to the findings of the Hisayama study, the proportion of high LDL was not so common in our study. One of the reasons for this might be due to the inclusion of large artery stroke in the form of an artery to artery embolic stroke, as the cardioembolic stroke was excluded from our study.

Our study showed a mean age of presentation (62.83±11.13 years) for acute non-cardioembolic ischemic stroke patients with dyslipidemia. This is in concordance with the study by Thapa A, et al. where the mean age of presentation among acute ischemic stroke patients in Nepal is almost similar (64.6±16.3 years).^9^ This is also supported by the findings from Feigin VL, et al. review showing a higher age-standardised incidence of stroke (4.2-11.7/1000 person-years) among those aged ≥55 years.^21^ The studies by Ovbiagele B, et al. (2011) and Brewer L, et al. respectively showed the likelihood of having a stroke nearly doubling every 10 years after the age of 55 years and a very high incidence of ischemic stroke (66%) among those aged ≥65 years.^22,23^ This greater liability of ischemic stroke among the elderly and aged population could be because of the increasing elderly population with an increase in the life expectancies due to the current prevention strategies. Likewise, the inevitable ageing-related cerebral and progressing atherothrombotic alterations occurring within the aged brain makes age one of the most important non-modifiable risk factors for acute ischemic stroke. In our study, a higher proportion of patients with dyslipidemia belonged to the ≥55 years age group (77.14%) which is contradicted by a study done by Zhao P, et al. showing a higher prevalence of deranged lipid profile among the non-elderly ischemic stroke patients.^24^ But, independent studies by Smith EE, et al. and Lu D, et al. reported dyslipidemia being more common among the elderly population, similar to the findings of our study.^25^’^26^ One reason for this discrepancy in findings could be because of the differences in the sample sizes and population definition of the studies. Likewise, the proportion of dyslipidemia in stroke patients in our study was higher in females (57.14%) as compared to males (42.86%). A study conducted in China reported low HDL cholesterol is more common among male patients while high LDL cholesterol, high TG and high TC are significantly more common among female patients which is similar to the findings in our study.^24^ The differences in the pattern of dyslipidemia in female and male patients might be due to the estrogen's effect, as studies have shown premenopausal women being less likely to suffer an ischemic stroke than postmenopausal women and men of the same age.^27-29^ Moreover, numerous genes on the second X chromosome are also found to affect ischemic stroke incidence, as shown by Carrel L, et al. study on X-inactivation profile.^30^

Though the findings from this study cannot be generalised owing to its small sample size and descriptive nature, it can still give us an insight into the overall burden of dyslipidemia among the acute non-cardioembolic ischemic stroke patients to help direct early interventions towards timely diagnosis, monitoring and treating dyslipidemia to prevent the stroke incidences.

## CONCLUSIONS

The prevalence of dyslipidemia among acute non-cardioembolic ischemic stroke patients at a tertiary care centre in Eastern Nepal is higher than the similar studies done in similar settings. The findings from this study imply that the intervention to curb the prevalence of non-communicable diseases such as stroke must be directed towards early diagnosis, monitoring and treating dyslipidemia with an emphasis on the primary level of prevention.
